# Molybdenum/Niobium Disilicide Multilayers Fabricated by Tape Casting: Microstructure, Mechanical Properties and Oxidation Behaviour

**DOI:** 10.3390/ma19081653

**Published:** 2026-04-21

**Authors:** Dreidy Mercedes Vásquez, Elisa Padovano, Claudio Badini, Sara Biamino, Luca Lavagna, Matteo Pavese

**Affiliations:** 1 Department of Applied Science and Technology, Politecnico di Torino, Corso Duca degli Abruzzi 24, 10129 Torino, Italy; dreidy.vasquez@pucv.cl (D.M.V.); elisa.padovano@polito.it (E.P.); claudio.badini@polito.it (C.B.); sara.biamino@polito.it (S.B.); luca.lavagna@polito.it (L.L.); 2 Escuela de Ingeniería Química, Pontificia Universidad Católica de Valparaíso, Av. Brasil 2950, Valparaíso 2362854, Chile

**Keywords:** molybdenum silicide, niobium silicide, tape casting, mechanical properties, oxidation resistance

## Abstract

MoSi_2_-based intermetallics are interesting materials for high-temperature applications, due to their moderate density, high melting point and significant oxidation resistance. In this paper, MoSi_2_-based materials in the form of multi-layered structures were fabricated by tape casting and pressureless sintering. Composites containing up to 20 wt.% of NbSi_2_ were produced, with the aim of obtaining biphasic structures with low pest oxidation at low temperature. The prepared samples were characterised with regard to phase composition, microstructure, mechanical properties and oxidation resistance. It was shown that the addition of a limited amount of NbSi_2_ prevents the pest oxidation phenomenon characteristic of pure MoSi_2_. Silica inclusions responsible for lowering the material toughness, were observed to disappear in the sintered silicides, thanks to the presence, during the binder burn-out, of a reducing atmosphere and to the carbonaceous residua. The phase and composition analysis also revealed the formation of small amounts of secondary phases like silicon carbide.

## 1. Introduction

The study of intermetallics is becoming more and more important because of the higher demand of aerospace and microelectronics industries for materials that can operate at higher temperature and with a better mechanical and chemical performance than currents alloys (e.g., nickel-based alloys, see for instance [1,2]). The interest is focused on the development of new materials with characteristics such as good fracture toughness, creep and fatigue resistance combined with resistance to oxidation or to a corrosive environment, in particular at high temperature. The modification of known materials is thus required to allow this properties improvement [1,2,3,4].

MoSi_2_-based materials are one of the possibilities currently envisaged in this field. Two main characteristics make molybdenum disilicide a potential material for use in applications subjected to severe thermal and corrosive environments: its high melting point (2030 °C) and its excellent oxidation resistance at high temperature, coupled with a relatively low density (6.20 g/cm^3^), high Young’s modulus at room temperature (440 GPa) and good thermal and electrical conductivity [5,6,7]. Its good thermal conductivity also suggests applications where an effective cooling of the products is needed, like engine components, substituting a forced cooling system [8]. Another interesting property of molybdenum silicide is its limited thermal expansion coefficient, between 8 and 10·10^−6^ K^−1^ from room temperature to 1400 °C [8].

MoSi_2_ is, however, a rather brittle compound. Its fracture toughness at room temperature is around 2–3 MPa m^1/2^, even if some papers report it to be up to 5 MPa m^1/2^ [9], depending of the testing technique used. Moreover, it has a brittle-to-ductile transition at high temperature (over 1000 °C) and both tensile strength and creep resistance decrease substantially with the increase in temperature over the brittle-to-ductile transition [10]. Apart from the inherent brittleness of the intermetallic compounds, another cause of low toughness of this compound is the presence of silica inclusions in the sintered parts [3,4,5,6,7]. Silica forms on the surface of the powders during their synthesis, and during the sintering step it segregates inside the material with the formation of inclusions that generate brittleness and worsen oxidation resistance.

At high temperature, MoSi_2_ presents an outstanding oxidation resistance, thanks to the formation of a dense SiO_2_ glassy film, which protects the underlying material from further oxidation. However, at intermediate temperatures, a phenomenon reported as “pest oxidation” occurs [11,12,13,14,15]. This is due to the formation of molybdenum oxide, following the equation:

2 MoSi_2_(s) + 7 O_2_(g) → 2 MoO_3_(s) + 4 SiO_2_(s) (1)

Molybdenum oxide forms at rather low temperatures (400–800 °C), but then it melts and starts evaporating, between 800 and 1200 °C, so that no passivation can occur. Only over 1200 °C does the oxidation follow a passivating route, since no molybdenum oxide forms, through the equation:

5 MoSi_2_(s) + 7 O_2_(g) →Mo_5_Si_3_(s) + 7 SiO_2_(s) (2)

Pest oxidation is favoured due to the presence of flaws inside the material, such as pores, internal cracks and SiO_2_ inclusions.

Thus, to promote the application of MoSi_2_ as a structural material in aerospace industry, it is necessary to improve both oxidation resistance and room temperature mechanical properties. In order to increase the mechanical properties of molybdenum disilicide, one of the possibilities is to use a second phase to modify the base material [16,17,18]. One of the promising candidates for this task is niobium disilicide, which presents similar thermal behaviour, high melting point and high strength at high temperature [19,20,21,22,23,24,25,26,27,28,29,30,31,32].

Another possible approach is to find a preparation technique for this compound that helps to improve the properties. The main preparation methods used for molybdenum disilicides are arc melting, mechanical alloying and combustion synthesis, even if other approaches are used, like shock synthesis, chemical vapour deposition or infiltration, reactive vapour infiltration and field-activated combustion synthesis [33,34,35,36]. For the consolidation of dense MoSi_2_ components, many approaches have been applied [33], including hot pressing, hot isostatic pressing, plasma spray, rapid solidification rate, low-pressure plasma spraying, vacuum plasma spraying, spark plasma sintering and tape casting, which is the one used in this work. Most of them, however, are based on the application of pressure at high temperature and thus are less versatile than pressureless sintering methods.

In this paper, we propose the tape casting technique, followed by pressureless sintering, using NbSi_2_ as a substitution of MoSi_2_. Since the substitution amount is small, NbSi_2_ acts almost as a sintering aid, but with the aim of improving oxidation resistance.

Tape casting is a method used to produce multilayer materials by the stacking of thin or thick layers [37,38,39,40,41]. The technique allows one to obtain parts with uniform density and homogeneous pore size distribution. It consists of preparing a well-dispersed suspension of ceramic powders, through the use of a dispersant agent, mixed with other organic components such as binders and plasticisers, and casting it on a polymeric support to obtain flat and thin sheets that are flexible and strong. The process is simple, industrially scalable and low-cost, and through a convenient stacking of single tapes, it is possible to produce components of various shapes and size. The most successful industrial use of this technique is in the electronic industry, and there is high potential in the fabrication of solid oxide full cells, amongst others.

In this paper, this technique is used because it can provide high strength to the prepared materials, as demonstrated by Biamino et al. [5], who successfully processed MoSi_2_ using tape casting followed by pressureless sintering. This paper indicates that this process seems also to reduce the formation of oxide inclusions in the silicide intermetallic, improving both strength and oxidation resistance. By the introduction of weak or porous layers in the multilayered structure, tape casting may also allow improvements in the toughness through crack deflection and the consequent increase in the dissipated energy before fracture [42].

Molybdenum silicide multilayers were also studied by Zhang and co-workers [43], who obtained by hot-pressing complex multilayers systems consisting of a sandwich structure of Al_2_O_3_, TiC and MoSi_2_ + Mo_2_B_5_ layers, where MoSi_2_ + Mo_2_B_5_ showed super-plastic behaviour. Tuffe et al. [44] proposed a MoSi_2_–Al_2_O_3_ structure in which the multilayer was constructed by an internal part of pure MoSi_2_ and an external one of a composite containing 25% of Al_2_O_3_. The material was obtained by hot pressing. Dumont and co-workers [45] proposed instead a MoSi_2_–Al_2_O_3_ functionally graded material fabricated by tape casting and sintering by SHS, showing a gradient in electrical conductivity through the thickness. Roncari et al. [46] worked on the AlN–SiC–MoSi_2_ system, obtaining densities of up to 94% after pressureless sintering aided by sintering aids, but showing no mechanical data. Chen et al. [47] used elemental powders of Mo, Si, and C and pressureless sintering, obtaining 93% density with a final content of 10% silicon carbide. Magnani et al. [15] instead used liquid infiltration of a porous preform to obtain a density higher than 92%. In these last cases, however, MoSi_2_ is used in an unalloyed form, thus not addressing the issues related to its oxidation resistance. Jo and Shon [48] proposed a 50%MoSi_2_–50%NbSi_2_ nanostructured composite obtained by a pulsed current-activated synthesis and consolidation method. They obtained a duplex phase with good density but the only mechanical property considered is hardness, together with an estimation of toughness from crack length. Kang and Shon [49] prepared also a 50%MoSi_2_–50%NbSi_2_ composite by high-frequency induction heated sintering, with results very similar to the ones obtained by Jo and Shon.

Several other research groups have prepared MoSi_2_-based composites: Zhang et al. [50] prepared a MoSi_2_–CNT (carbon nanotube) composite by hot pressing, obtaining very good toughness for a 6% CNT content. A similar material was also recently realised by Nazari et al. [51]. More recently, Zhang et al. [52] prepared a MoSi_2_–UHTC composite, using ZrB_2_ and SiC, for heating element applications. For the same application, Wick-Joliat et al. [53] prepared a MoSi_2_–Al_2_O_3_–feldspar composite by ceramic-injection moulding, but in this case, MoSi_2_ was present only for providing conductivity, and the main component was the other ceramics. Feng et al. [54] produced some Si_3_N_4_–MoSi_2_ composites with La_2_O_3_ and Y_2_O_3_ sintering aids by hot pressing, where the main contribution to strength was given, however, by the Si_3_N_4_ phase, while Titov et al. [55] used Si_3_N_4_ as an additive to improve low temperature oxidation of MoSi_2_ obtained by hot pressing. More recently, Demir et al. [56] realised a MoSi_2_–MoB_2_ composite with improved fracture toughness, and Huang et al. [57] a MoSi_2_/Al_2_O_3_ composite with interesting strength. The most recent publication is by Bei et al. [58], who proposed a MoSi_2_–MoAlB composite with high strength and toughness. In all these cases, the use of composite materials allowed improvements in toughness and strength.

Slightly different materials were prepared by Gao et al. [59], who produced some interesting MoSi_2_–RSiC interpenetrating phase composites, albeit with low strength, while similar composites were produced by Xie et al. [60], using phenolic resin infiltration-pyrolysis and MoSi_2_–Si–Ti alloy-activated melting infiltration. Silicon infiltration was used by Huang et al. [61] to produce dense SiC_f_/MoSi_2_ composites, but multiple impregnation–calcination cycles are needed to have a continuous MoSi_2_ phase, and residual Si is present. Zhang et al. [62] too prepared a nano MoSi_2_–SiC composite by melt infiltration of silicon.

Another interesting approach is also from Jain et al. [63], where laminated composites between MoSi_2_/SiC and a metal (Mo, Ta, Nb) were realised. More recently, Kaledin et al. [64] fabricated layered SiC/C/Si/MeSi_2_/Me composites via liquid silicon infiltration. Lu et al. [65,66] prepared MoSi_2_–SiC composites by vacuum hot pressing, with good mechanical properties and improved pest oxidation resistance by pre-oxidation at 1200 °C of MoSi_2_ containing Nb, Al and SiC. Monteverde et al. [67] prepared MoSi_2_–ZrB_2_ materials with dual composite architecture by hot pressing, while Pogozhev et al. [68] prepared ZrB_2_–ZrSi_2_–MoSi_2_ and HfB_2_–HfSi_2_–MoSi_2_ composites by magnesiothermic SHS and hot pressing. A similar technique was used by Gorshkov et al. [69] to produce (MoW)Si_2_. Yeh and Peng [70] produced MoSi_2_–Al_2_O_3_ with thermite-like reactions in a wide range of compositions, while Zaki et la. [71] used a similar reactive approach to obtain mullite/MoSi_2_ composites.

Regarding oxidation resistance, Potanin [72] prepared MoSi_2_–HfB_2_–MoB composites, observing the formation of a borosilicate oxide surface layer and also of HfSiO_4_, with good oxidation results. Borosilicate glass was used by Tao et al. [16] to guarantee self-healing ability to a MoSi_2_/borosilicate glass composite. Vorotilo et al. [73,74] used MoSi_2_–MoB composites obtained by SHS and hot pressing with the idea of limiting MoO_3_ evaporation at medium temperature through the formation of a borosilicate glass. Yan et al. [75] also used boron (working in the Mo–Si–B system) to improve oxidation resistance in SPS composites, observing improved performance with increasing B content. Zhang et al. [76] prepared in situ MoSi_2_-SiC-MoB composites with low pest oxidation but not optimal mechanical properties. Safaie et al. [77] added Al to consume SiO_2_, while Silvestroni [78] studied the oxidation of SiC/MoSi_2_ composites at ultra-high temperature. Recently, Huang et al. [79] suggested the use of WSi2 as an additive to MoSi_2_ for improved oxidation resistance.

Several studies concern the use of MoSi_2_-based composites as coatings for oxidation protection of C/C composites or Mo- or Nb-based alloys. Hu et al. [80] prepared MoSi_2_–mullite coatings for C/C composites protection, while Chen et al. [81] prepared MoSi_2_–SiC composites for graphite protection by using recycled MoSi_2_ heating elements. Li et al. [82] prepared MoSi_2_/(Mo,Ti)Si_2_ dual-phase composites to protect Mo alloys, and finally, Bezzi et al. [83] developed SiC/MoSi_2_ composites as C/C coatings. Recently, Zhu et al. [84] prepared a MoSi_2_ coating with SiC whiskers for oxidation protection, while Zhang et al. [85] proposed the superposition of a pre-oxidised (Nb,X)Si_2_ layer with a MoSi_2_ coating to improve adhesion and oxidation resistance. An interesting approach was also proposed by Ji et al. [86], who used thermal expansion mismatch to design gradient coatings of ZrB_2_, MoSi_2_ and a borosilicate glass. In all these cases, the oxidation was performed at very high temperature, thus not considering the effect of pest oxidation. Zhai et al. [87] proposed a ZrB_2_/SiC/MoSi_2_ coating that exploited rapid oxidation at low temperature as a way to reduce pest oxidation issues.

This expansive literature suggests that the topic of MoSi_2_-based composites is currently of great interest for the scientific community, and oxidation resistance remains one of the key factors to consider when studying these kinds of materials.

The aim of this paper was to study the oxidation resistance of MoSi_2_-based materials when coupling the preparation by the tape-casting technique with the addition of small quantities of NbSi_2_. The use of tape casting allows the elimination of silica inclusions, as observed in a previous paper of ours [5], thus improving oxidation resistance. The addition of NbSi_2_ in small quantities (up to 20 wt.%) should guarantee a finer duplex microstructure. Phase composition, mechanical properties and oxidation resistance (at low and high temperature) were analysed for these composites.

## 2. Materials and Methods

The powders of molybdenum and niobium silicide were obtained by ABCR. The mean powder size for MoSi_2_ was 2–3 μm, while the NbSi_2_ powders were larger, with a wide distribution under 45 μm. In the powders, two impurities were observed: SiO_2_ (on the surface) for both powders, and Mo_5_Si_3_ or Nb_5_Si_3_ for MoSi_2_ and NbSi_2_, respectively.

**Figure 1:**
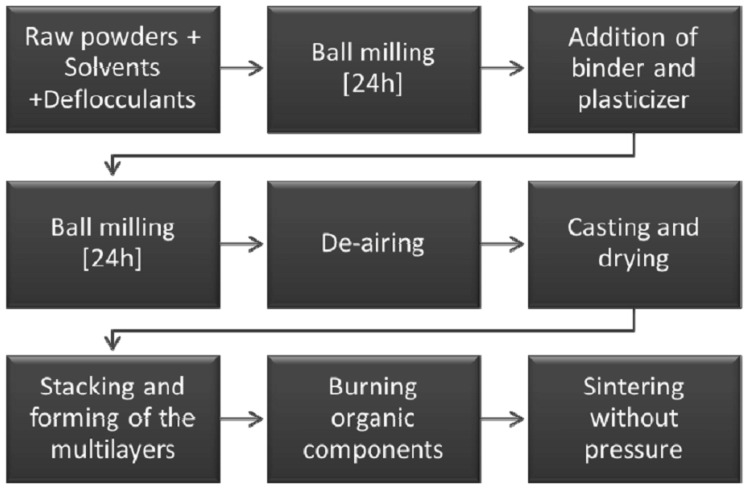
Flow chart of the preparation method of the multilayers by tape casting and pressureless sintering.

As discussed in the introduction, tape casting is a technique that involves the combination of different components into the slurry to provide a very well-mixed material for the fabrication of flat sheets. The flow chart of the preparation method is shown in Figure 1. The powders were dispersed in inorganic solvents by the addition of 0.1% fish oil and then mixed for 24 h in a ball mill. After the addition of binder and plasticiser, further mixing for 24 h was performed. The slurries were rheologically optimised in a preliminary step of the work, in order to obtain a viscosity and a rheological behaviour suitable for the subsequent casting step. The detailed composition of the slurry is given in Table 1. During the ball-milling step, air can be entrapped into the slurry, so that before casting, a step of vacuum degassing was performed, keeping the slurry in a vacuum chamber until no more bubbles were observed on the surface (around 15 min). The casting was carried out on a tape casting machine with a stationary doctor blade and a moving Mylar^®^ carrier film. All the slurries were cast at 100 mm/min of speed and the blade gap was adjusted to 1 mm. The cast slurry was slowly dried in calm air at room temperature to eliminate the organic solvents, giving a flexible sheet of thickness around 200–250 μm.

The multilayer samples were prepared by cutting the green dry tapes in rectangular pieces of 60 × 100 mm, and stacking the pieces one upon the other, making sure to put in contact a rough surface (the one in contact with the air during casting) with a smooth one (the one in contact with the Mylar support during casting). By using a glue and by rolling with a mandrel, every layer adhered perfectly to the underlying one, thus avoiding the formation of air bubbles between layers. The bond glue was prepared by mixing water, ethanol and polyvinyl alcohol (PVA). For every composition, eight multilayer samples of 60 × 12 mm with 10 layers were fabricated by cutting one 60 × 100 mm rectangular multilayer.

**Figure 2:**
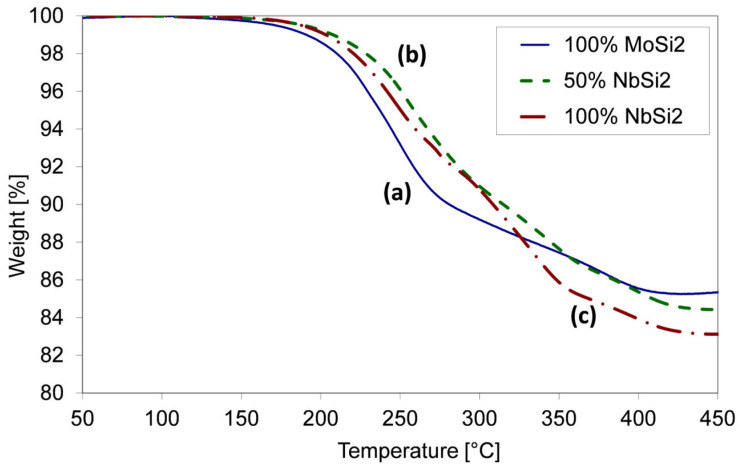
Thermogravimetric curves, under argon flow at the heating rate of 1 °C/min, of tapes containing: (a) 100%MoSi_2_, (b) 50%MoSi_2_–50%NbSi_2_, (c) 100%NbSi_2_.

Binder, plasticiser and other additives were burned out by slow heating up to 800 °C in an Elite oven in a flowing argon atmosphere to prevent oxidation and to carry away the products of the decomposition of the organic substances. In order to determine the optimal heating rate for this step, the organics decomposition was studied by thermogravimetric experiments realised on dried tapes. A Mettler Toledo TGA/SDTA 851e (Greifensee, Switzerland) instrument was used, with a measurement accuracy of 0.5 K and a resolution of 1 μg. The thermal decomposition of a tape containing MoSi_2_ powder in argon flow at the slow heating rate of 1 °C/min is depicted in Figure 2, together with the decomposition behaviour of tapes containing mixed MoSi_2_–NbSi_2_ powders (50%MoSi_2_) or pure NbSi_2_ powder. Most of the weight is lost between 200 and 400 °C, where the thermal decomposition of binder and plasticiser occurs. The difference in the decomposition profile of the tapes containing silicides of different Mo and Nb contents are not very significant, and can be probably ascribed to a catalytic effect of the metals on the decomposition kinetics.

The debinding treatment was chosen based on the curves shown in Figure 2 and on previous experience [5,88,89]. An isotherm at 70 °C for 10 h had the function of completing the evaporation of solvent, while a very slow heating rate, 0.25 °C/min, was used from 70 °C to 800 °C. The very slow heating had the function of avoiding the fast evolution of gases, which could cause the formation of bubbles, cracks and defects in the green ceramic. The maximum temperature was chosen as 800 °C, in order to provide an acceptable mechanical resistance to the specimens that will be transferred to the sintering oven.

Pressureless sintering was performed in a Pro.Ba. graphite furnace (Cambiano, Italy) at a temperature between 1600 and 1800 °C for 30 to 60 min. Based on these preliminary experiments, in this paper, we report only the results for the 1725–1785 °C range and 30 min sintering time, where the best results were obtained, and further characterisation was performed. The heating and cooling rates were 6 °C/min and the atmosphere was argon at a pressure of 600 mbar. During sintering, the samples were immersed in a mixture of powders of SiC (75 wt.%) and NbSi_2_ (25 wt.%) inside graphite boxes.

The characterisation of the sintered samples included the analysis of the microstructure and of the crystalline phases, the measurement of density, mechanical properties and oxidation resistance. The geometric density of multilayers was measured in the green state, after debinding and sintering. In order to distinguish the open and closed porosity, apparent density of the sintered samples was also measured, using Archimede’s method with water as a fluid. Picnometry was used to determine the theoretical density of MoSi_2_ and NbSi_2_. Young’s modulus was measured on parallelepiped samples according to ASTM C 1259-21 [90] by using an impulse excitation technique, involving the analysis of the transient natural vibration (GrindoSonic MK5 Instrument, Leuven, Belgium), while Vickers microhardness (HV) was performed with a load of 500 g and a dwelling time of 10 s. Three-point bending strength was measured according to UNI EN 658.3 standard [91] (Sintech10D equipment, Shakopee, MN, USA), with a crosshead speed of 0.1 mm/min in stroke control and 40 mm span. For microscopy observation, rectangular samples were cut from the bending samples, mounted in transparent acrylic resin and polished down to 1 μm with diamond paste, and then cleaned in an ultrasonic bath with a mixture of water and ethanol, and subsequently etched to reveal the microstructure using a 20%vol HF, 40%vol HCl and 40%vol HNO_3_ attack for 5 min, as reported by Nakano et al. [28]. The microstructure of the samples was assessed by scanning electron microscopy (SEM-FEG Assing SUPRA 25, Oberkochen, Germany), chemical composition by energy-dispersive spectroscopy (EDS Oxford, High Wycombe, United Kingdom), and phase composition by X-ray diffraction (Philips PW1710 Cu_Kα_ radiation, Almelo, Netherlands). Thermogravimetric experiments were realised with a Mettler TG/SDTA 851e (Greifensee, Switzerland, performing runs in a controlled atmosphere (either air of argon) up to 1600 °C, at a rate of 10 °C/min.

## 3. Results and Discussion

### 3.1. Microstructure, Composition and Densification

In this work, NbSi_2_ was used in a composition range between 5 and 20 wt.% of niobium disilicide, even if some samples were realised at high NbSi_2_ content. The rationale behind this choice is that the structure with the most promising properties is the duplex one, where both MoSi_2_ and NbSi_2_ phases are present. Thermodynamic calculations demonstrate that the two silicides can be in pseudo-binary equilibrium [80], and this thermodynamic stability allows the formation of a duplex structure, due to the linkage of the crystallographic parameters of C11b (MoSi_2_) and C40 (NbSi_2_) structures, in the 5–20 wt.% NbSi_2_ range [32]. For this reason, we investigated mainly the 5–20 wt.% of niobium disilicide, using pure MoSi_2_ as a reference and higher NbSi_2_ content samples to verify our assumption regarding the phases present after sintering.

**Figure 3:**
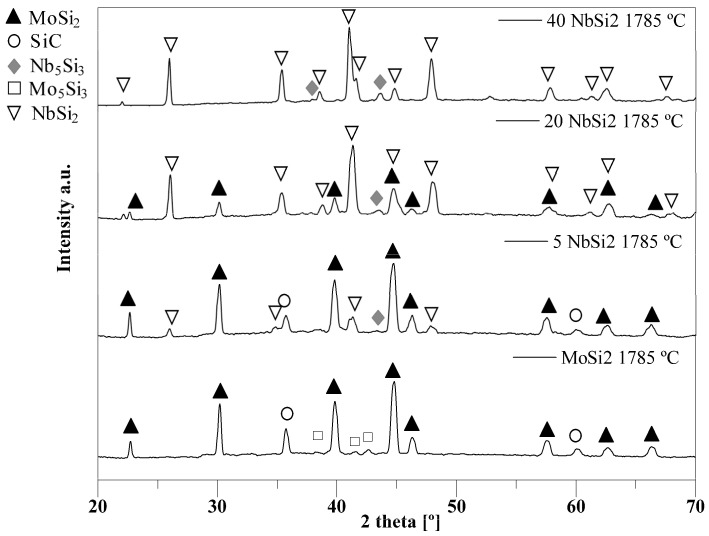
XRD spectra of MoSi_2_–NbSi_2_ multilayers sintered at 1785 °C: pure MoSi_2_, 5 wt.% NbSi_2_, 20 wt.% NbSi_2_, 40 wt.% NbSi_2_.

In Figure 3, X-ray diffraction spectra of the inner portion of the samples fabricated at 1785 °C are presented, where the presence of the duplex structure is confirmed when NbSi_2_ is added. The range 5–20 wt.% observed by Geng et al. [92] for the formation of duplex structures is confirmed by this analysis, while further increasing the niobium disilicide content up to 40 wt.% brings it to the single C40 phase, which is the same as with pure NbSi_2_. The presence of other secondary phases, SiC, Mo_5_Si_3_, Nb_5_Si_3_, can be also observed, either present in the starting powders as impurities or formed in situ due to the reaction of the two disilicides with the carbon residua left by the organic components during the burning out.

In the case of pure molybdenum disilicide, these secondary phases are SiC and Mo_5_Si_3_. A Rietveld refinement of the spectrum suggests around 35% of SiC and less than 5% of Mo_5_Si_3_ phase, which presents low peaks in the X-ray pattern but whose presence was confirmed by the EDS analysis. This phase probably derives from impurities in the powder (which is known to contain a small amount of the 5-3 silicide). Another possible source for the formation of Mo_5_Si_3_ is the reaction of MoSi_2_ with carbon, which is widely study in the literature because the addition of carbon is one of the strategy routes to reduce or avoid the formation of the silica glassy phase generally observed in these compounds due to the high quantity of oxygen left around the particles of MoSi_2_ and NbSi_2_ during the powder-preparation step [3,4,5,6,7,93]. These SiO_2_ inclusions were most often reported to be the cause of lower mechanical properties. In the presence of carbon, however, the literature suggests that not only is Mo_5_Si_3_ formed, but also Mo_5_Si_3_C, or Mo_4.8_Si_3_C_0.6_, which belong to the class of Nowotny phases [5,93,94]. No such phase is observed in this case, so the origin of Mo_5_Si_3_ was ascribed only to impurities in the powders.

By increasing the niobium content, Mo_5_Si_3_ is no longer seen, while Nb_5_Si_3_ peaks are observed. Moreover, silicon carbide content decreases with niobium increase. A hint of NbC phase is observed in some cases, but the attribution is unclear. Rietveld refinement of the spectra suggests that in the sample containing 5 wt.% NbSi_2_, the silicon carbide content decreases to under 30%, while a small quantity of NbSi_2_ appears (around 10%). When increasing niobium disilicide to 20 wt.%, silicon carbide disappears, and the main phase becomes NbSi_2_, at around 75%, with only traces of Nb_5_Si_3_, at around 1%, the rest being MoSi_2_. In the sample containing 40 wt.% NbSi_2_, the MoSi_2_ phase disappears, and only NbSi_2_ (around 90%) and Nb_5_Si_3_ remain.

**Figure 4:**
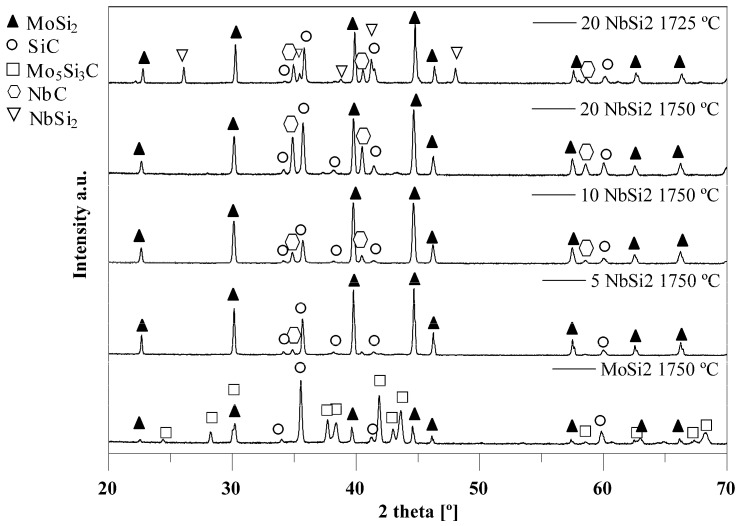
XRD pattern of the surface of MoSi_2_-NbSi_2_ multilayers, sintered at 1750 °C (pure MoSi_2_, 5 wt.%NbSi_2_, 10 wt.%NbSi_2_, 20 wt.%NbSi_2_) and at 1725 °C (20 wt.%NbSi_2_).

To better investigate the reason for the absence of Nowotny phase in the samples, XRD was also performed on the surface, where the carbon content is higher, probably due to the interaction with the oven atmosphere, and the results are shown in Figure 4, where the reported samples sintered at 1725 and 1750 °C.

In this case, the observed secondary phases are SiC, the Nowotny phase Mo_5_Si_3_C, and NbC. For pure MoSi_2_, it is evident that with a higher carbon content, SiC and Mo_5_Si_3_C are formed. In particular, Rietveld refinement suggests less than 10% MoSi_2_, around 50% of Nowotny phase and 45% of SiC.

With increasing NbSi_2_ content, SiC quantity remains more or less constant, while NbC appears. Rietveld refinement suggests that NbC is present as 2–3% in the case of the sample containing 5% NbSi_2_, increasing to around 5% in a 10% NbSi_2_ sample, and to almost 15% in a 20% NbSi_2_ one. In the literature, the most widely reported secondary products formed in the materials containing niobium disilicide are Nb_5_Si_3_C, and Nb_5_Si_3_. However, some evidence of NbC formation is seen, for instance, by Yaney et al. [95], who observed the formation of NbC as a primary reaction product when they studied Nb–Si–C–O reaction systems. In their investigation, it was reported that high quantities of oxygen and carbon in the reaction seems to promote the formation of NbC. In the ternary phase diagram of the Nb–Si–C system, they reported that while NbSi_2_ and SiC can be in equilibrium, the presence of the Nb_5_Si_3_ phase promotes the formation of NbC. Thus, the presence of Nb_5_Si_3_ in this system can also promote the formation of niobium carbide.

Moreover, on the surface, no C40 (NbSi_2_) phase is observed even in the case of niobium-rich compositions, for the samples sintered at 1750 and 1785 °C. At 1725 °C, only with 20 wt.%NbSi_2_ the C40 phase is observed (between 15 and 20%), while at lower Nb concentration there is only the C11_b_ phase (MoSi_2_). These results suggest that a higher carbon content pushes the reaction toward the Nowotny phase in the case of pure MoSi_2_, and toward NbC in the case of NbSi_2_-containing samples, consuming the niobium and leaving the C11_b_ phase as the predominant one. Instead, at low carbon content, i.e., in the interior of the samples, only a small amount of SiC is formed, and the two silicides are rather stable, with the coexistence of the C40 and C11_b_ phases. Another important observation is that with Nb substitution, no Nowotny phase is observed. This confirms that niobium suppresses the formation of Mo_5_Si_3_C phase, promoting instead the formation of NbC.

**Figure 5:**
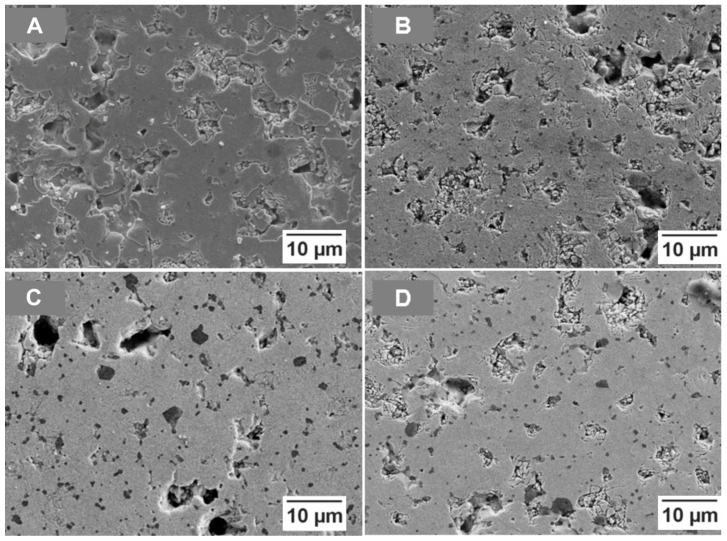
Backscattered electrons SEM images of multilayers sintered at 1725 °C for 30 min: (**A**) MoSi_2_; (**B**) 95%MoSi_2_–5%NbSi_2_; (**C**) 90%MoSi_2_–10%NbSi_2_; (**D**) 80%MoSi_2_–20%NbSi_2_.

**Figure 6:**
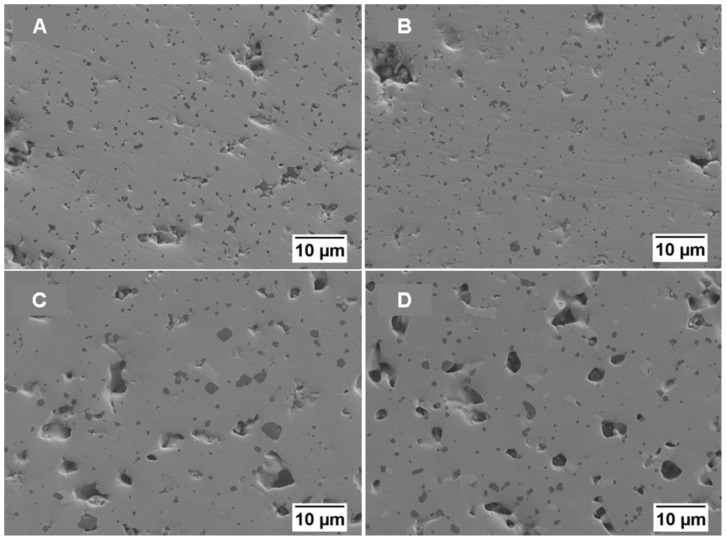
Backscattered electrons SEM images of multilayers sintered at 1750 °C for 30 min: (**A**) MoSi_2_; (**B**) 95%MoSi_2_–5%NbSi_2_; (**C**) 90%MoSi_2_–10%NbSi_2_; (**D**) 80%MoSi_2_–20%NbSi_2_.

**Figure 7:**
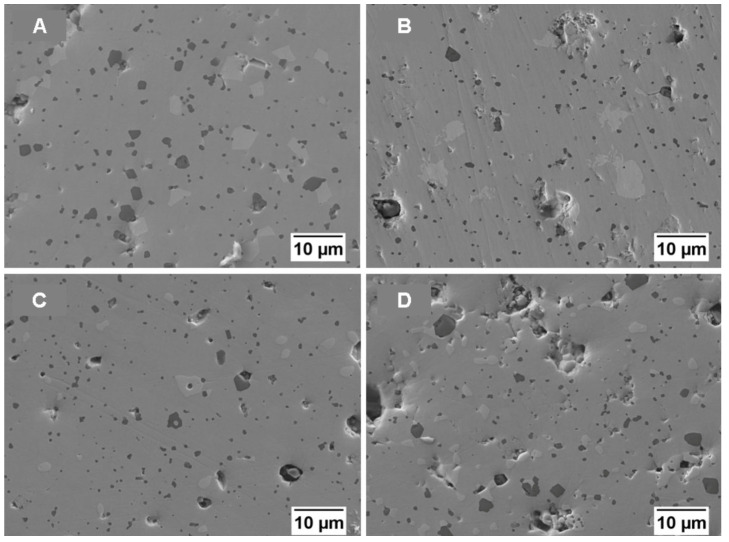
Backscattered electrons SEM images of multilayers sintered at 1785 °C for 30 min: (**A**) MoSi_2_; (**B**) 95%MoSi_2_–5%NbSi_2_; (**C**) 90%MoSi_2_–10%NbSi_2_; (**D**) 80%MoSi_2_–20%NbSi_2_.

The microstructure of the materials sintered at the three different temperatures (1725, 1750 and 1785 °C) is reported in Figure 5 and Figure 6 and 7, respectively. Case (A) represents pure MoSi_2_, case (B) 5%NbSi_2_, case (C) 10%NbSi_2_, and case (D) 20%NbSi_2_. These images are taken with backscattered electrons, so the different phases exhibit different shades of grey. MoSi_2_ is the medium grey phase, SiC the dark phase (since it has the lowest mean atomic number) and Mo_5_Si_3_ or Nb_5_Si_3_ are the brighter phase (since they have the highest mean atomic number). In these images, it is not possible to distinguish between molybdenum and niobium, since their atomic number is very similar. Umakoshi, Nakano and co-workers in their investigations were able to see a difference between the two silicides in SEM images only when they annealed the samples to obtain a lamellar structure, and only with compositions with 10 and 15 wt.% of NbSi_2_ [24,25,26,27,28,29,30,31,32]. In this case, EDS was used instead to measure the relative Mo and Nb content. It must also be mentioned that at 1725 °C, the different phases are not very easily distinguished, due to the smaller grain size and to the high residual porosity content evidenced in Figure 5. In particular, the Mo_5_Si_3_ or Nb_5_Si_3_ phase begins to be clearly observed only at 1750 °C.

At 1725 °C, for pure MoSi_2_, the SiC grains are mostly extremely small, in the submicrometric range, and are well-distributed in the matrix material, helping to control the growth of MoSi_2_ grains. As already anticipated in the discussion of XRD results, the main reason of the formation of this phase is the reaction of carbon from the organic component of the slip with silicon oxide or molybdenum disilicide, following these reactions:

SiO_2_ + 3 C → SiC + 2 CO (3)

5 MoSi_2_ + 7 C → Mo_5_Si_3_ + 7 SiC (4)

5 MoSi_2_ + 8 C → Mo_5_Si_3_C + 7 SiC (5)

The SiO_2_ into the MoSi_2_ matrix in some reports is located at the grain boundaries, wetting the MoSi_2_ grains, and in others, within the grains; other works found the silica inclusions in both locations [3,4,5,6,7,93]. The in situ formation of SiC seems to have the same behaviour, irrespective of where the SiO_2_ is positioned. If the silica is in the grain boundary, the SiC will be there, and the same will happen when it is located inside the grain. Thus, the silicon carbide grains appear well-distributed among the MoSi_2_ ones. The predominant mechanism of SiC formation is probably given by reaction (3), since no Nowotny phase is observed by XRD and the Mo_5_Si_3_ phase is already present in the powders. It is not possible to exclude the contribution of reaction (4), while, contrary to what is suggested in the literature, reaction (5) was not observed.

For samples containing NbSi_2_, it is possible to note how SiC grains increase in size with respect to the pure MoSi_2_ case, reaching the micron range for 10 wt.% and 20 wt.%NbSi_2_-containing samples.

Increasing the temperature (to 1750 °C and 1785 °C, Figure 6 and Figure 7, respectively) allows for grain growth, and the secondary phases (SiC, Mo_5_Si_3_ or Nb_5_Si_3_, and NbC in the case of high niobium content) become more evident. In Table 2, the image analysis results, in terms of area-weighted distribution of SiC particle diameter, are reported for the samples sintered at 1750 and 1785 °C. The correspondence with the SEM images is clear, suggesting that the smaller SiC grain size is observed for the samples containing 5%NbSi_2_.

In detail, at 1750 °C, both in the pure MoSi_2_ and in the 5 wt.% NbSi_2_ samples it is still observed a large fraction of the submicrometric SiC phase (d50 is smaller than 1 μm). Instead at 10 wt.% and 20 wt.% NbSi_2_ a larger amount of micrometric SiC grains is observed, with also a slight formation of NbC, as also confirmed by EDS analysis. The 5-3 silicide grains are typically larger than the SiC ones, generally over 1 μm, but at 1750 °C, they are observed only with 10% or 20%NbSi_2_.

At the highest temperature used, 1785 °C, the microstructure presents a larger grain size both for the secondary phases and for the silicides. In all compositions, the presence of Mo_5_Si_3_ or Nb_5_Si_3_ silicides was observed even when not clearly observed by XRD analysis. Again, the size of 5-3 silicides was over 1 μm, while no significant differences in the grain size distributions were observed as a function of niobium silicide content. The SiC grains remain instead rather small, even if they are larger than at 1750 °C in the pure MoSi_2_ case.

As already anticipated, a very interesting behaviour of all the samples, both of pure molybdenum disilicide and containing niobium, was that no silica glassy phase was ever observed after sintering. The reason for this very positive effect must probably be searched in the use of tape casting as the processing method; the silica reduction process probably starts during the debinding phase, and ends during the sintering thanks to the small carbonaceous residua left from the decomposition of binder, plasticiser and other organic components of the slurry. Thus, there is no need to add carbon directly to the powders, as it is performed with other techniques, like hot pressing. As described in the discussion of Figure 4, avoiding the addition of an excessive quantity of carbon should also be beneficial in terms of phase formation, since lower amounts of brittle Nowotny phase, SiC and NbC are formed.

**Figure 8:**
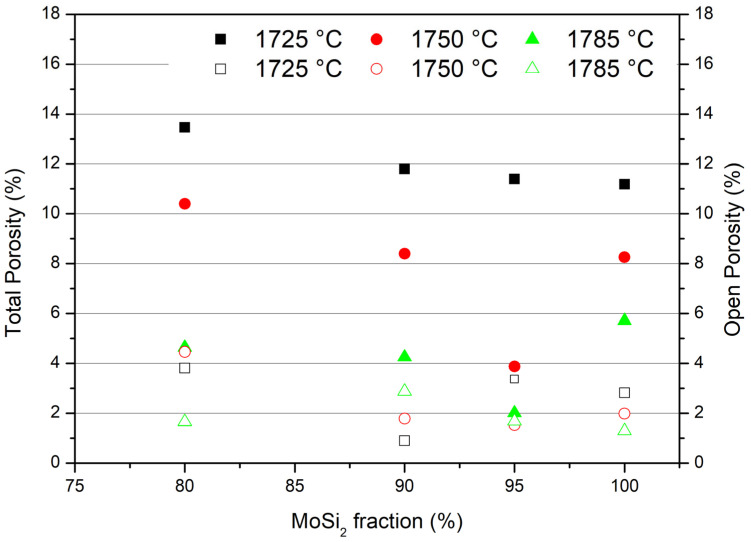
Total porosity (filled markers) and open porosity (unfilled markers) of MoSi_2_–NbSi_2_ sintered multilayers at 1725 °C (■,□) 1750 °C (●,○) and 1785 °C (▲,△).

The open and total porosity data for the MoSi_2_–NbSi_2_ multilayers are shown in Figure 8, where filled markers represent total porosity and unfilled markers represent open porosity. Total porosity data demonstrate that an increase in temperature causes a better densification of the materials, as confirmed by the microstructures (Figure 5, Figure 6 and Figure 7), where a reduction in the porosity is observed at a higher temperature, even if the pores can grow due to the coalescence of the small ones. A smaller and more uniform pore distribution, even if the number of pores is greater, is present in the samples sintered at 1725 °C.

Similar results were obtained by Biamino et al. [5] on multilayers of pure MoSi_2_, where the relative density of samples sintered at 1700 °C and 1750 °C were around 85 and 88%, respectively. It must be also considered that the samples of pure MoSi_2_ of the Biamino paper were sintered using carbon as protective powder, while in this case, the samples were immersed in a mixture of powders of SiC and NbSi_2_ inside graphite boxes in order to limit the possible reaction of niobium disilicide with carbon. By limiting the reaction with carbon, an increase in the final density was observed. Some tests performed with a higher sintering time (60 min) suggested that the increase in sintering time did not provide further benefit in densification.

Regarding the effect of niobium silicide introduction, the addition of a small quantity of NbSi_2_ (5%) caused an enhancement in densification in the multilayers, at 1750 and 1785 °C; with respect to pure MoSi_2_, the total porosity is only 4% and 2%, respectively. When the fraction of NbSi_2_ increases further, the density goes down again, and densification values similar to the pure molybdenum disilicide are found. At 1725 °C, the increase in niobium silicide content causes a small reduction in the density.

Open porosity is also reported in Figure 8 because it is important for oxidation behaviour (see Section 3.3). The trend is unclear; however, open porosity is generally higher for the samples with low overall density and for those sintered at higher temperature.

### 3.2. Mechanical Properties

**Figure 9:**
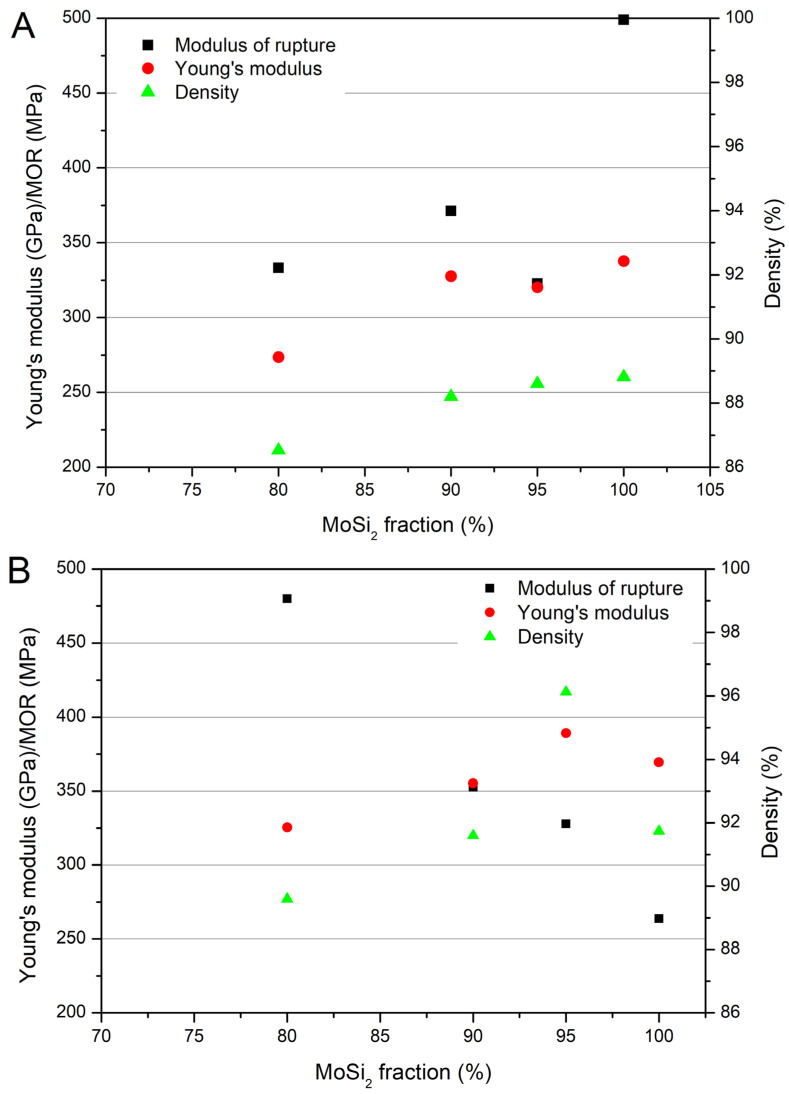

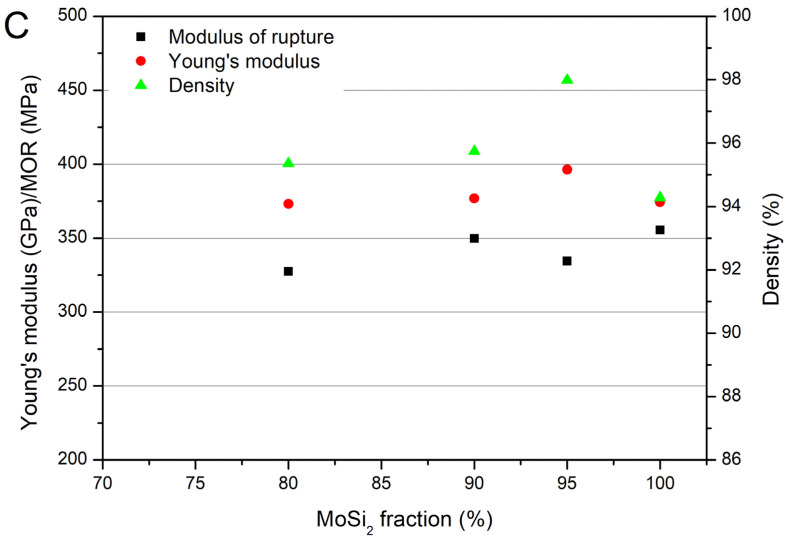
Relative density (▲, right-hand *y* axis), Young’s modulus (●, left-hand *y* axis) and modulus of rupture (MOR) (■, left-hand *y* axis) of the multilayers sintered at (**A**) 1725, (**B**) 1750 and (**C**) 1785 °C as a function of MoSi_2_ content.

**Figure 10:**
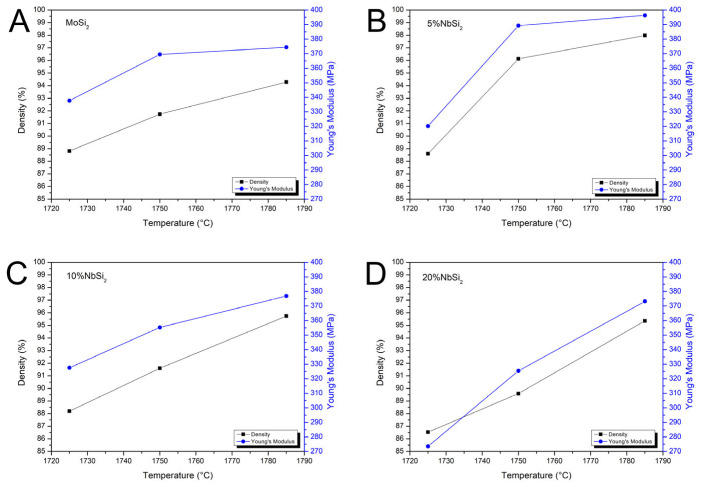
Relationship between relative density (■) and Young’s modulus (●) as a function of temperature for the different compositions: (**A**) MoSi_2_, (**B**) 5%NbSi_2_, (**C**) 10%NbSi_2_, and (**D**) 20%NbSi_2_.

The trend observed for density is generally clearly reflected in the elastic modulus, for all the studied compositions. In Figure 9, data for relative density (diamond symbol), Young’s modulus (triangle symbol) and modulus of rupture (square symbol) are plotted against the MoSi_2_ fraction for the three sintering temperatures. There are no significant differences between the trend of the values of the Young’s modulus and the one of the density, with the lowest values observed for high NbSi_2_-containing samples and low sintering temperature. If the sintering temperature increases, as already observed in the density case, the Young’s modulus increases up to close to 400 GPa. This behaviour is shown more clearly in Figure 10, where the correlation between Young’s modulus and density is presented as a function of sintering temperature for the different studied compositions.

At a sintering temperature of 1725 °C, the minimum addition of 5 wt.% NbSi_2_ (Figure 10B) had a negligible effect on the density but reduced the Young’s modulus compared to the pure MoSi_2_ (Figure 10A). However, at higher sintering temperatures, the most substantial increase in both density and Young’s modulus was observed. A further increase in the NbSi_2_ content (10 wt.% and 20 wt.%) leads to a more pronounced decrease in the material’s density, particularly at low (1725 °C) and medium (1750 °C) temperatures, compared to pure MoSi_2_ (Figure 10C,D). Conversely, at the highest sintering temperature of 1785 °C, all samples with 10 wt.% NbSi_2_ and 20 wt.% NbSi_2_ exhibited a marginally improved density and a comparable Young’s modulus.

Regarding the modulus of rupture/bending strength, a complex behaviour is observed. It is interesting to observe both the strength versus sintering temperature and the strength versus NbSi_2_ fraction trends.

In the first case, with pure MoSi_2_, the small size of pores and grains observed for the samples sintered at 1725 °C allows for higher bending strength (up to almost 500 MPa), while at higher temperatures, the mechanical resistance is reduced due to the increase in the size of both pores and secondary brittle phases. This behaviour is similar to the one observed in [5], where the increase in density and Young’s modulus was accompanied by a decrease in bending strength. The decrease is not linear with temperature probably because two phenomena are occurring at the same time: reduction in strength due to the increase in pore and grain size, and increase in strength due to the increase in density. At a sintering temperature of 1725 °C, the addition of NbSi_2_ causes a reduction in bending strength with respect to pure MoSi_2_, with values ranging between 320 and 370 MPa. This reduction can be partially related to the density, but also to the increase in the size of the silicon carbide precipitates, as observed in Figure 5, Figure 6 and Figure 7. Also the phase composition could play a role, with the opposite behaviour of the brittle NbC phase, reducing strength, and of the duplex structure, which can increase the mechanical properties of MoSi_2_–NbSi_2_ materials [25,27,29,31,32,96].

**Figure 11:**
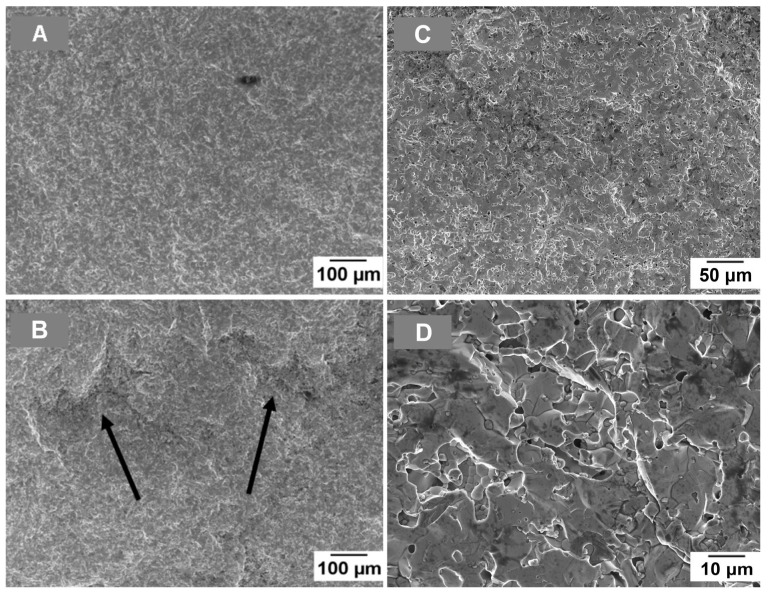
Fracture surface of samples sintered at 1750 °C: (**A**) pure MoSi_2_, (**B**) MoSi_2_–20%NbSi_2_, (**C**,**D**) higher magnification images of the interlayer zone; black arrows in (**B**) indicate the interface between different layers.

The samples sintered at 1750 °C present a different behaviour with respect to those sintered at 1725 °C. It is evident from Figure 9 that the addition of NbSi_2_ has a positive effect in this case, and the samples containing 20 wt.% of NbSi_2_ present a very high strength, 480 MPa. To explain this phenomenon, two causes can be considered. The first concerns the crack deflection mechanism at the interfaces between different layers. The tapes containing high amounts of niobium silicide, in particular the 20 wt.% NbSi_2_ one, present a higher surface roughness with respect to the pure MoSi_2_ ones, probably due to the presence of the fraction of the larger-sized NbSi_2_ powder in the slurry. The tapes are superimposed so that a rough surface adheres to a smooth one (the one in contact with the Mylar sheet); the rougher the tape, the higher the porosity level at the interface between each of the layers of the multilayer. In Figure 11, it is possible to observe the fracture surface of 20 wt.% NbSi_2_ sample (Figure 11B) compared with a pure MoSi_2_ one (Figure 11A). The samples are well-sintered, save for the interlayer zone marked with black arrows in Figure 11B, where long interconnected pores are present. These porous zones can promote crack deflection, as happens in layered structures with porous interlayers or weak interfaces [32,97]. In Figure 11C,D, a magnification of the porous zone evidenced by the black arrows is presented, in order to better show the porous zone that facilitates crack deflection. The other possible cause of the increase in the strength of samples sintered at 1750 °C with niobium silicide content is related to the presence of the duplex structure, where the C11b phase act as a minority reinforcement for the C40 phase, as shown by XRD data and confirmed by the literature [31,32,96]. This type of structure was suggested to be effective in strengthening the material [25,27,29], since the C11b phase was shown to prevent the initial rapid propagation of cracks, causing crack deflection and improving ductility.

**Figure 12:**
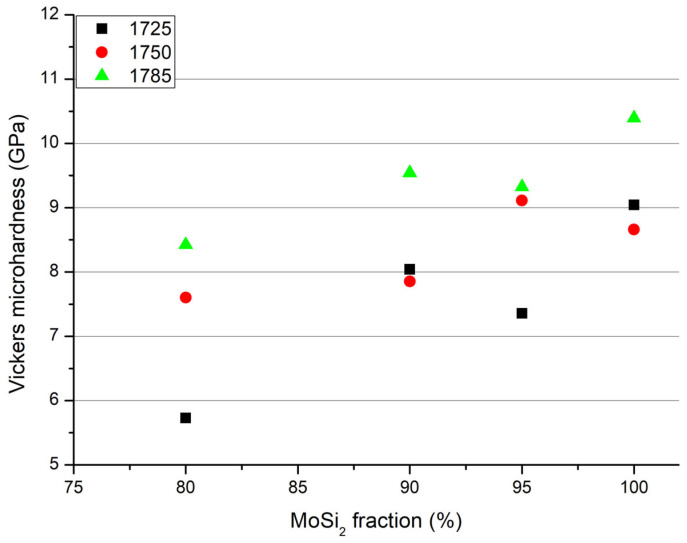
Vickers microhardness of samples sintered at 1725 °C, 1750 °C and 1785 °C, as a function of NbSi_2_ content.

The microhardness behaviour is also shown in Figure 12. The hardness increases with the sintering temperature, i.e., with the density, and decreases in the presence of NbSi_2_. The presence of niobium silicide negatively affects the hardness, probably due to the presence of the brittle NbC phase.

### 3.3. Oxidation Behaviour

**Figure 13:**
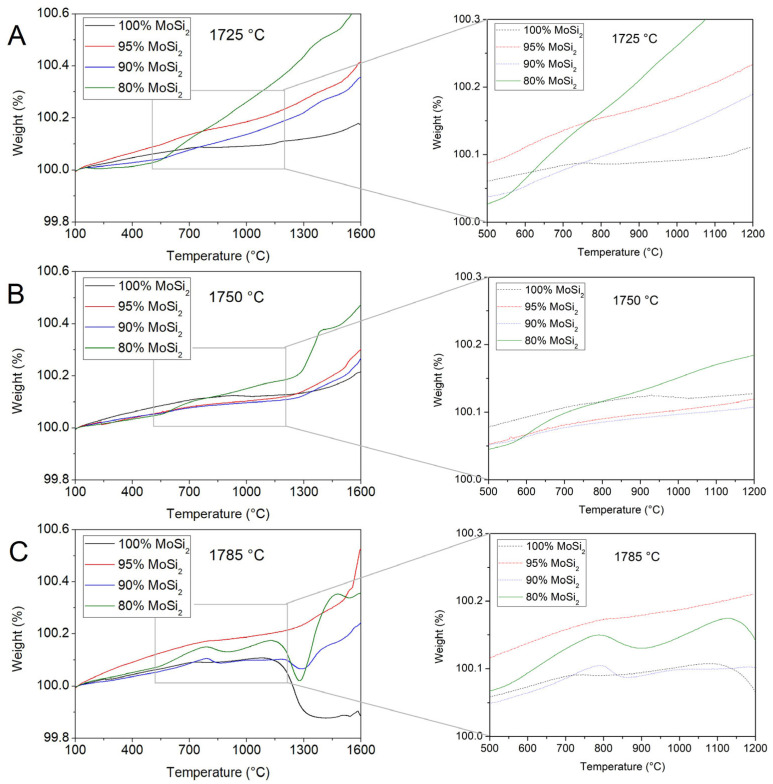
TGA curves of samples sintered at 1725 °C (**A**), 1750 °C (**B**) and 1785 °C (**C**), for samples with different MoSi_2_ content. On the right side, a magnification of the zone between 500 and 1200 °C, the pest oxidation region, is presented.

As already mentioned in the introduction, MoSi_2_ has a great potential as a structural material for application at high temperature due to its outstanding oxidation resistance at high temperature, but the issues connected with the pest oxidation must be overcome [11,12,13,14,15]. In this study, the oxidation behaviour of the MoSi_2_–NbSi_2_ materials was realised through thermogravimetric experiments. In Figure 13, TGA curves of the multilayers of all the compositions are reported for samples sintered at 1725, 1750 and 1785 °C.

In general, when analysing oxidation curves, three important characteristics of the material must be kept into account: first, the composition, which is the main driver to the oxidation resistance; second, the open porosity of the samples, which determines the amount of surface accessible to the oxidising atmosphere, and thus the overall weight gain; third, the grain size, which will determine the homogeneity of the oxidised layer: if the grains are small, the layer will have more uniform composition and less residual stresses at the interfaces. If the grains are large, spalling of the oxidised layer will be easier.

The curves of samples obtained at 1725 and 1750 °C are rather similar (Figure 13A and Figure 13B, respectively). The pure MoSi_2_ samples (black curves) show a limited weight increase at high temperature, demonstrating a good passivation behaviour, but present a plateau in the curve between 700–800 °C and 1100–1200 °C. The plateau is due to the balance between MoSi_2_ oxidation and MoO_3_ evaporation, and its presence confirms pest oxidation behaviour [12,13,14,75,98]. With the addition of small quantities of niobium disilicide, the plateau is not observed anymore, and the weight grows steadily with the temperature. This suggests that a change in the oxidation mechanism occurs, as observed also by several authors for boron addition [12,75,76]. Too high a NbSi_2_ quantity, however, is not beneficial to the oxidation resistance, for two reasons. First, Nb_2_O_5_ can be formed upon oxidation [99,100,101,102,103], which can have several effects. It can reduce the compactness of the oxide layer, thus causing higher oxidation rates. Additionally, it is known to cause spallation of the oxidised coating due to significant volume changes [103]. Finally, it can form elongated grains [100,102] that reduce the cohesion of the layer. Second, NbC is also present, and it can generate internal cracks when the ratio of the NbSi_2_–Nb_5_Si_3_–NbC–SiC is not appropriated [95], since it can act as nucleation site for the oxidation mechanism.

The samples sintered at 1785 °C (Figure 13C) present complex curves that could be interpreted as taking into account the bigger mean grain size, and thus less homogeneity of the layer, and the higher open porosity (as shown in Figure 9, except for the 20 wt.% NbSi_2_ samples). In particular, spalling phenomena can cause a sudden weight increase, while evaporation causes a plateau of or a gradual decrease in weight in the curve. Also at this temperature, the best sample is the one containing 5 wt.% NbSi_2_, which does not present evident MoO_3_ evaporation or spalling phenomena.

**Figure 14:**
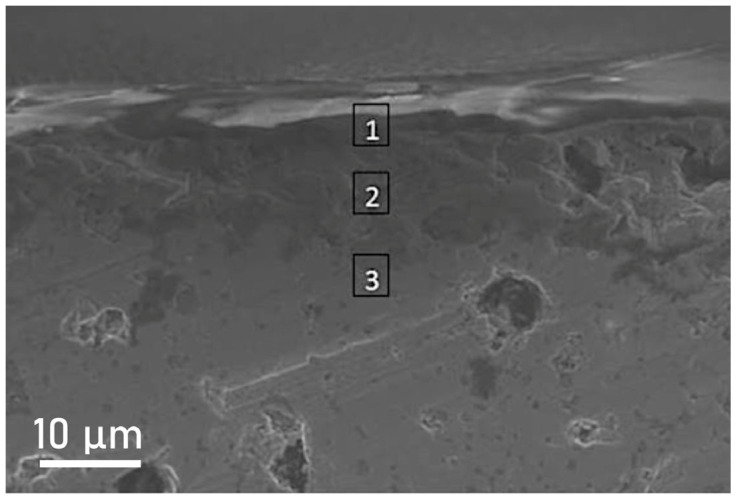
SEM image of the sample with 5 wt.% NbSi_2_ sintered at 1750 °C.

The morphology after oxidation of the sample with 5 wt.% NbSi_2_ sintered at 1750 °C, which has the best oxidation behaviour, is depicted in Figure 14. SEM observation demonstrated the presence of two layers close to the surface: the underlying MoSi_2_ is indicated with the number 3; a region is then observed (number 2) containing SiO_2_ and molybdenum silicides (both MoSi_2_ and Mo_5_Si_3_); and finally, a dense layer of pure silica (number 1) that avoids the further oxidation of the sample.

From all these data, it is possible to try to draw some conclusions. First, it seems evident that the sintering temperature must be strictly controlled to guarantee, at the same time, a proper densification and limited secondary reactions. If, at 1785 °C, the density, Young’s modulus and microhardness are higher, it is clear that oxidation resistance decreases. The formation of larger grains probably reduces the ability of the material to properly withstand oxidation without spalling of the oxide layer.

Regarding the optimal amount of NbSi_2_, the data suggest that 5% or 10% are the best cases. From a mechanical point of view, pure MoSi_2_ or 20%NbSi_2_ can give very good results, but pest oxidation is present in the case of pure MoSi_2_, while a too-high oxidation rate is observed when 20% NbSi_2_ is used, probably due to the formation of a layer with reduced protective ability.

The reason for this behaviour can be inferred by crossing the information of XRD and microstructural observations. Diffraction spectra show that a duplex microstructure is obtained when a small quantity of NbSi_2_ is added; moreover, using only 5% of NbSi_2_ allows a better densification at both 1750 and 1785 °C, with higher density and Young’s modulus, and a limited grain growth. The samples with 10% NbSi_2_ have higher porosity and grain size but comparable mechanical properties. The density and microstructure observed for the samples sintered at 1750 °C allows for the best oxidation resistance, which requires an absence of pest oxidation, as also observed by Huang and coworkers with WSi_2_ [79]. These authors discuss in detail the effect of WSi_2_ on the oxidation resistance of MoSi_2_–20%Al_2_O_3_ composites, concluding that it is due to two factors: the limited diffusion of Si atoms when W is present, and the consequent limited thickness of the oxide layer, which limit the rupture and peeling of the oxide layer. It must be stressed, however, that these authors use a MoSi_2_–20%Al_2_O_3_ composite, and that their oxide layer also contains Al_2_O_3_ in a mullite form. In this case, the presence of a duplex structure could work in a similar way, hindering the diffusion of silicon, but the presence of NbSi_2_ probably also has the effect of limiting MoO_3_ formation. A similar effect was observed by Li et al. [104], who added chromium to MoSi_2_ to obtain a duplex structure between C40 and C11b, as in this case, claiming an increase in oxidation resistance.

In conclusion, it seems that the best samples are those containing 5 wt.% or 10 wt.% NbSi_2_, sintered at 1750 °C, where the best coupling of mechanical properties (modulus or rupture and Young’s modulus) and oxidation resistance is obtained.

## 4. Conclusions

In this work, molybdenum disilicide multilayers were prepared by the tape-casting technology followed by pressureless sintering, studying the effect of the addition of a small fraction of NbSi_2_ on densification, microstructure, mechanical properties and oxidation resistance. The results show that a small amount (5 wt.%) of niobium silicide can improve the density, Young’s modulus and oxidation resistance of MoSi_2_, even if the flexural strength is reduced. The sintering temperature had a significant effect on the properties, and it was shown that it is important to find an equilibrium between lower sintering temperature, which results in higher porosity but smaller pores and less intensive reactions between the components, and higher sintering temperature, which reduces the porosity but results in an increase in the pore size and in the open porosity, thus causing higher stiffness but lower strength and lower oxidation resistance. The best compromise between strength, stiffness and oxidation resistance was found for 5 wt.% NbSi_2_ at 1750 °C sintering temperature, where good values for stiffness (350 GPa) and flexural strength (>300 MPa) were obtained, together with the best oxidation resistance and no evident pest oxidation. Moreover, the silica glass inclusions that form during sintering are reduced during the processing route thanks to the products of the decomposition of the organic components of the slurry necessary for the tape casting.

## Figures and Tables

**Table 1:** Slurry composition for the different multilayers produced in the MoSi_2_/NbSi_2_ system.

Components		MoSi_2_	5%NbSi_2_	10%NbSi_2_	20%NbSi_2_	40%NbSi_2_	50%NbSi_2_	NbSi_2_
wt. % MoSi_2_		100	95	90	80	60	50	0
Solvents	Ethanol	14.8						
	Butanol	22.8						
Dispersant	Fish oil	0.1						
Powders	MoSi_2_	51.4	48.8	46.3	41.1	30.8	25.7	0.0
	NbSi_2_	0.0	2.6	5.1	10.3	20.6	25.7	51.4
Binder	Polyvinylbutyral	7.2						
Plasticiser	Polyethyleneglycol	3.7						

**Table 2:** Image analysis results on SiC phase for the MoSi_2_/NbSi_2_ materials sintered at 1750 and 1785 °C: grain size distribution parameters.

Sintering *T*	1750 °C				1785 °C			
Sample	MoSi_2_	5%NbSi_2_	10%NbSi_2_	20%NbSi_2_	MoSi_2_	5%NbSi_2_	10%NbSi_2_	20%NbSi_2_
d10	0.45	0.4	0.52	0.55	0.7.	0.4	0.45	0.55
d50	0.9	0.75	1.5	1.2	1.5	0.85	0.95	1.4
d90	1.6	1.2	3.4	2.2	3.3	1.5	2.5	3.8

## Data Availability

The original contributions presented in this study are included in the article. Further inquiries can be directed to the corresponding author.
